# Teaching Magnetoelectric Sensing to Secondary School Students—Considerations for Educational STEM Outreach

**DOI:** 10.3390/s21217354

**Published:** 2021-11-05

**Authors:** Cara Broß, Carolin Enzingmüller, Ilka Parchmann, Gerhard Schmidt

**Affiliations:** 1 IPN—Leibniz Institute for Science and Mathematics Education at Kiel University, 24118 Kiel, Germany; parchmann@leibniz-ipn.de; 2 Institute for Electrical Engineering and Information Engineering, Kiel University, 24143 Kiel, Germany; gus@tf.uni-kiel.de

**Keywords:** public understanding/outreach, ME sensors, medical sensing, biomagnetic sensing, interdisciplinary/multidisciplinary

## Abstract

A major challenge in modern society is the need to increase awareness and excitement with regard to science, technology, engineering and mathematics (STEM) and related careers directly or among peers and parents in order to attract future generations of scientists and engineers. The numbers of students aiming for an engineering degree are low compared to the options available and the workforce needed. This may, in part, be due to a traditional lack of instruction in this area in secondary school curricula. In this regard, STEM outreach programs can complement formal learning settings and help to promote engineering as well as science to school students. In a long-term outreach collaboration with scientists and engineers, we developed an outreach program in the field of magnetoelectric sensing that includes an out-of-school project day and various accompanying teaching materials. In this article, we motivate the relevance of the topic for educational outreach, share the rationales, objectives and aims, models and implementation strategies of our program and provide practical advice for those interested in outreach in the field of magnetoelectric sensing.

## 1. Introduction

Attracting motivated and skilled workers in science, technology, engineering and mathematics (STEM) is difficult [1]. Adding to this problem, the ability to combine knowledge and skills from STEM disciplines will be increasing in demand as more and more institutions recognize innovations in integrated STEM areas as the key to the future economy and social progress [2,3,4]. Fostering these interdisciplinary competencies and skills takes time and careful planning. Often enough, schools do not have the capacity to fully prepare their students in this regard, as they traditionally focus on subject knowledge [5]. Furthermore, students’ interest in certain STEM subjects tends to be low and continues to decline in developed countries [6], and misconceptions and stereotypes of science, scientists and engineers are rather common among students [7,8]. As a consequence, many school students do not want to pursue careers in these fields.

One way to address these issues could be by rethinking the way STEM is taught: Research indicates that STEM education can be a promising approach to strengthening students’ abilities to combine knowledge and skills across disciplines as well as fostering their interest in STEM disciplines [2,5,9]. STEM education promotes the integration of the four disciplines, science, technology, engineering and mathematics, actively combining knowledge and competencies across disciplines within one context. Ideally, examples of authentic research and representations of researchers in the respective fields are integrated with STEM contents [10,11]. Combining the ideas of STEM education and authentic representations, university-led outreach initiatives can provide an excellent opportunity to deliver STEM education based on real-world interdisciplinary problems and authentic insights into modern science and engineering [9,12,13]. In doing so, they can complement formal school education and foster interdisciplinary skills and interest in STEM.

In this paper, we present a design-based STEM-integrated outreach activity centered around magnetoelectric sensing within the context of heart diagnostics. Besides STEM content, the program provides authentic insights into the interdisciplinary work of scientists and engineers. It was developed in collaboration with teachers, school students and researchers of the field of biomagnetic sensing and is based on our professional educational experience. The target group for the outreach activity consists of upper secondary school students in grades ten through thirteen (age fifteen and older) with prior knowledge in biology, chemistry and physics at the lower secondary level who have chosen the science profile as their A-level program.

## 2. Magnetoelectric Sensors for Medical Applications

### 2.1. Biomagnetic Sensing in the Collaborative Research Centre “Magnetoelectric Sensors”

Biomagnetic sensing is the measurement and analysis of magnetic fields of living organisms [14]. Such magnetic fields are induced by the same ionic currents that generate bioelectrical fields by flowing in and between cells. Biomagnetic sensing can therefore be used to monitor and analyze electrophysiological processes, such as cardiac electrical activity.

The most common method of measuring cardiac electrical activity is electrocardiography. Since its introduction well over a century ago, it has been optimized to derive information such as the rhythm of heartbeats or damage to the heart muscle from electrical potential differences on the body surface [15]. It was only in the last few decades that biomagnetic fields were discovered as a valuable source of medical information, introducing corresponding measurement approaches in the 1960s and 1970s [16]. These contactless measurements avoid inaccuracies due to poor electrical contact or the inhomogeneous conductivity of bodily tissue, which affect the electrical fields’ propagation. They also have a higher spatial resolution and the possibility of providing sensors with better positioning, which leads to fewer exogenous signal artifacts [17].

However, biomagnetic signals are typically weak and can easily be polluted by environmental magnetic noise. The heart’s magnetic field strength lies at amplitudes lower than 100 pT (which is about 500,000 times smaller than the earth’s magnetic field’s amplitude) and at frequencies between 0.01 and 100 Hz [14,17]. Currently, biomagnetic signals such as the heart’s magnetic field can be measured with complex sensor technology such as superconducting quantum interference devices (SQUID) [18] or optically pumped magnetometers (OPMs) [19]. SQUID sensors require liquid cooling to operate and necessitate a magnetically shielded environment. OPMs are non-cryogenic but also require magnetic shielding for their use in medical contexts such as magnetocardiography. Both the necessary cooling and the magnetic shielding lead to high acquisition and operating costs and currently prevent mobile use.

**Figure 1:**
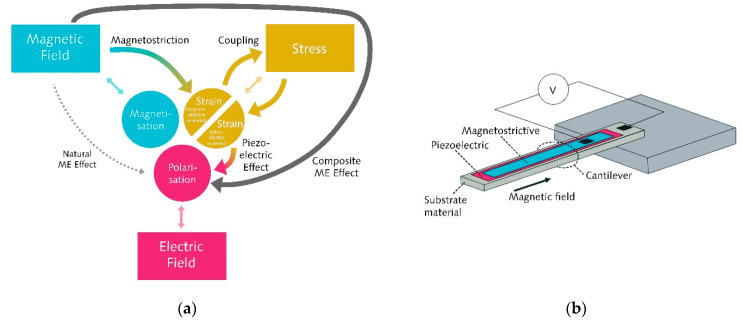
(**a**) Schematic representation of the composite ME effect and the natural ME effect. In this representation, only the direction that is used for magnetoelectric sensing is illustrated; (**b**) illustration of a ME cantilever beam sensor.

The Collaborative Research Centre 1261 “Magnetoelectric Sensors” (short: CRC 1261) works on the development and evaluation of sensitive, low-cost and uncooled magneto-electric (ME) sensors for medical contexts such as magnetocardiography (MCG) and magnetoencephalography (MEG). In contrast to SQUID sensors, which are made of single-phase magnetoelectric material, these new types of sensors are based on ME composites containing magnetostrictive and piezoelectric layers. By coupling the magnetostrictive strain to the piezoelectric phase (Figure 1), these sensors show a strong ME effect. One of the sensor principles that is the subject of research within the CRC 1261 is the bending beam principle.

**Figure 2:**
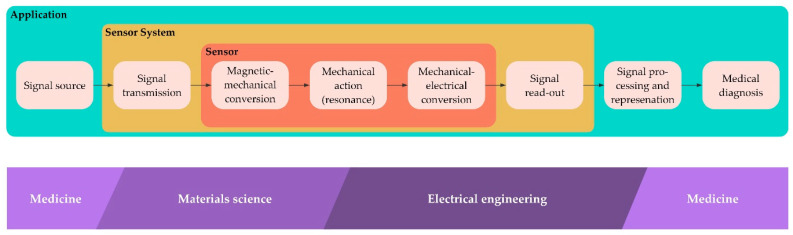
Biomagnetic sensing as a highly interdisciplinary application of magnetoelectric sensors (based on [20]).

The cantilever-type sensor is based on several measurement principles—the bending beam principle being one of them—combining knowledge of different disciplines (Figure 2). The sensor consists of a thin silicon layer as a substrate material on which at least two layers are applied: a piezoelectric and a magnetostrictive layer (Figure 1). Exposed to a magnetic field, the cantilever-type sensor bends similarly to a bimetal due to the magnetostrictive material. The bending of the sensor can be amplified using the sensor’s (mechanical) self-resonance. Approaches from information technology (i.e., modulation and demodulation) can then compensate for the frequency shift involved in the effect amplification.

### 2.2. Potential of Biomagnetic Sensing as a Topic for Outreach

STEM outreach is a form of science communication in which STEM institutions such as universities communicate findings and methods from current scientific research to the general public or schools. The goals of these outreach activities are often to increase STEM literacy, to foster interest in STEM and to promote positive attitudes towards STEM [12,21]. Biomagnetic sensing provides an excellent basis for STEM outreach activities, as it not only offers an ideal connection between students’ personal frame of reference and interdisciplinary STEM content, but also is perfectly suited for the representation of authentic research.

#### 2.2.1. Fostering Interdisciplinary Knowledge and Skills

Apart from fostering scientific knowledge within the separate STEM disciplines, bio-magnetic sensing provides a context for fostering interdisciplinary knowledge and skills. In our modern society, there is an increasing demand for the ability to connect knowledge and skills from different disciplines, not only in the professional domain, but also in everyday life [2,3]. In order to prepare students for this demand, students have to be explicitly taught to build and use interdisciplinary skills and knowledge. A field of educational research that strives to achieve this is STEM education (e.g., see [9]). Its main objective is to actively link STEM subjects to one another and to combine knowledge across disciplines. Often, this interdisciplinary work is motivated by solving interdisciplinary problems or is embedded into contexts that require interdisciplinary actions such as health, biodiversity, or climate change. Research showed that this makes it possible to teach the skill of integrating knowledge across disciplines. Furthermore, successful integrated STEM education can foster knowledge within and interest in the separate disciplines [1,9]. 

Context-based learning provides a suitable framework for teaching and learning in this regard. Instead of teaching lists of information which seem to be irrelevant and disconnected for many students, contexts provide authentic problems to be explored for which knowledge is necessary and skills have to be further developed. This approach has been widely investigated and shows positive results, especially with regard to the interests of students [22].

In order to successfully integrate STEM subjects, a suitable context is crucial. On the one hand, the context has to be interesting and relevant to students, and on the other hand, its complexity has to correspond with the students’ knowledge [2]. Biomagnetic sensing provides such a context. First, the medical context can illustrate the importance of basic physical-technical research for one’s own life and society. Studies have repeatedly shown that medical and human biological contexts are seen as particularly attractive to a diverse group of students and can be used to generate interest and increase the perceived relevance of learning content [23,24,25]. Secondly, the aim of developing sensors for medical applications creates an authentic demand for interdisciplinary collaboration, e.g., in the form of knowledge integration processes that are necessary when designing an effective medical sensor. This further motivates the learning of interdisciplinary skills and knowledge.

#### 2.2.2. Authentic Insights into the Nature of Science and Engineering

To understand the relevance and approaches of science for societal development, insights into what science is and how scientists and engineers work—concepts which are called the Nature of Science or Nature of Engineering, respectively [10,11]—are important in addition to content knowledge and skills. They prepare students to make informed decisions about the communication and application of science in their own lives and help to provide realistic experiences with STEM research that may result in them wanting to pursue a career within these fields [7]. Frameworks such as Programme for International Student Assessment (PISA) therefore incorporate the “knowledge about science” as well as the “knowledge of science” as elements of scientific literacy [26]. However, studies have shown that students often have a limited understanding of science and engineering as a way of knowing [10,27]. Learning about the nature of science requires students not only to simply engage in science and engineering activities, but also to explicitly address and discuss the nature of science and nature of engineering. That is to say, aspects of science or engineering are to be brought up in appropriate learning situations and illustrated by and discussed on the basis of those learning situations [11]. By doing so, one can convey insights into the inner workings of the respective disciplines, including questions such as “what is the nature of scientific knowledge?” or “how do engineers conduct their work?”

In the case of biomagnetic sensing research, interdisciplinary work is essential to developing a functional ME sensor suitable for medical applications. Portraying this interdisciplinarity not only allows one to discuss similarities and differences between disciplines such as materials science and electrical engineering, but also to highlight collaboration as an important feature of modern research. Studies show that social and cooperative components of modern research may have the potential to stimulate interest in science, especially among girls [28].

In summary, ME sensors and the context of biomagnetic sensing are very well suited to inspiring STEM learning. They can help students make connections across STEM disciplines and can be used to enrich knowledge about the nature of science and engineering through relevant contexts, interesting scientific principles that can be linked to the school curriculum, a variety of opportunities to integrate STEM disciplines, and the proximity to current research that allows portraying researchers and their work authentically.

## 3. Methods of Design

When designing educational interventions, tools and materials, a common and well-suited method is the so called design-based research approach [29]. Design-based research is led by two objectives [30]: first, the development of an effective practical intervention (e.g., an outreach event or learning materials), and second, the acquisition of theoretical insights concerning teaching and learning processes. It usually consists of an iterative process of design, implementation and evaluation [29].

In this outreach project, we used design-based research to first develop an out-of-school project day on biomagnetic sensing at a student laboratory, the Kiel Science Factory (https://www.forschungs-werkstatt.de/english/, accessed on 15 October 2021). An examination of the scientific content and the frame conditions such as students’ prior knowledge and interests, an exploration of the student lab as well as ideas and feedback from CRC researchers formed the basis for the design of a prototype project day. Our goal was to develop and test a learning environment that provides insights into the interdisciplinary field of biomagnetic sensing involving scientists and engineers working in that area. From an educational point of view, the design framework applied the goals and principles of context-based learning and integrated STEM education by contextualizing the project day within a medical problem, by developing experiments that allow hands-on activities and by integrating media elements that directly showcase the work of the scientists and engineers. The focus for the accompanying research was to better understand students’ interest and understanding in the science and scientists in the field of bio-magnetic sensing so that the design could be refined through several cycles. The major challenge in designing appropriate activities was to bridge the gap between traditional school science topics and the advanced content of the CRC. Explanations and hands-on experiments illustrating aspects of a real research process were required. 

**Figure 3:**
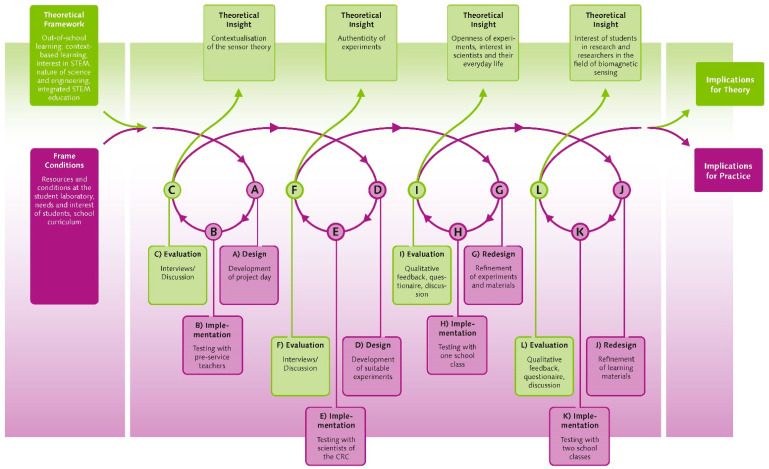
Illustration of the design-based research process that lead to the design of the project day.

The whole process involves several iterations of design, implementation and evaluation to develop the project day (Figure 3). In a first step, the CRC scientists were involved in the process of developing prototype tasks and materials to ensure the relevance, correctness and authenticity of the content. We held a series of meetings with CRC scientists to discuss initial ideas and presented our progress regularly at retreat meetings in front of the entire CRC. The CRC scientists not only provided their expertise and feedback on teaching materials and experiments, but also actively contributed ideas that enriched the design. One example of this was the co-development of an experiment (see Box 1), a process that also led to a joint publication in a physics education journal [31].

After developing a prototype for the whole project day, we again consulted different experts: we invited pre-service teachers as experts for levels and structures of learning environments and doctoral students of the CRC as experts for research practices in the area of biomagnetic sensing to test and evaluate the project day. The pre-service teachers gave feedback on the didactic structure and the instructional tasks at the learning stations, while the doctoral students gave feedback on the authenticity of the materials and experiments. We refined the project day, taking into account feasibility, educational quality and authenticity based on the feedback received.

After these expert consultations, we tested the project day with our main target group, upper secondary school students. For this, we invited three classes (*N* = 46, grade 12–13) and their science teachers to our student laboratory. They had between four and seven years of physics experience and between two and five years of chemistry experience in school. A short questionnaire was designed to assess comprehensibility and attractiveness of the project day. Additionally, students were asked to write down what they liked, what they missed and what they learned during the project day. A final iteration with larger cohorts of students and statistical analyses of the effects on interest, insights into the nature of science and engineering, and an understanding of the content had been planned but could not yet be realized, due to the COVID-19 Pandemic.

## 4. Results of the First Design Process

**Figure 4:**
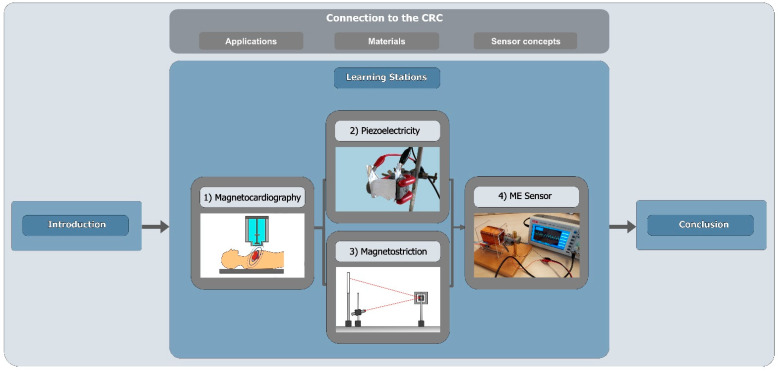
Structure of the project day at the Kiel Science Factory.

The current design of the project day at the Kiel Science Factory is based on the research logic of the CRC 1261, so that the students can follow and understand the development of ME sensors for medical applications (Figure 4).

Introduction. At the beginning of the project day, the students get to know the medical context of heart diagnostics in order to capture their interest and increase their perception of relevance [23,25]. This is followed by a presentation of the objectives of the CRC 1261, which shows the benefits and added value of ME sensors for heart diagnostics. Both students and teachers confirmed that they enjoyed this introduction, which embedded the ME sensing approach in a medical context. After this introductory phase, the students begin to work in small groups with their own supervisor—university students that received content instruction beforehand—in order to encourage an active exchange regarding the respective sensor principles or concepts. In addition, this group setting ensures a high level of feedback, which further fosters the motivation and interest of the students and facilitates STEM learning [1,32]. This was also confirmed by the feedback of the students. They particularly appreciated the opportunity to ask questions at any time. In these small groups, the students go through four learning stations, at the end of which they build their own ME sensor based on information and introductions into the underlying principles.

Station 1: Magnetocardiography. At the first station, the supervisors explain the underlying principles of the electrophysiological activities of the heart as the basis for cardiac diagnostics. Students then apply this knowledge, record a classmate’s electrocardiogram and learn to interpret it, drawing connections to their biology knowledge. Then, they are handed supplementary information on magnetocardiography and its advantages and disadvantages. Based on the knowledge acquired at this station, they compare the two diagnostic methods, electrocardiography and magnetocardiography. 

Stations 2 and 3: Piezoelectricity and Magnetostriction. At the following two stations, the students learn about piezoelectricity and magnetostriction as sensor material properties. Based on a model explanation of the piezoelectric phenomenon by the supervisor, students develop an experimental setup to measure the piezoelectric effect of piezoelectric crystals grown from Rochelle salt at station 2. For this purpose, they are provided with various materials, including piezoelectric crystals, aluminum foil as an electrode, a multimeter, cables, crocodile clips, different electrically isolating materials and stand equipment, which they can use for their experimental setup. They compare and discuss their results before moving on to station 3. Analogous to station 2, the supervisor of station 3 first explains the magnetostrictive phenomenon. Students then apply their knowledge by conducting a model experiment in which the magnetostrictive effect is made visible with the help of a magneto-optical sensor (for more information, refer to Box 1). Both stations with a focus on sensor materials were very well received, and the hands-on nature of these stations was highlighted particularly positively. Based on the feedback, we incorporated different levels of scaffolding—supporting students in acquiring knowledge—to allow students with different prior knowledge and skills to conduct their own inquiry.

**Figure 5:**
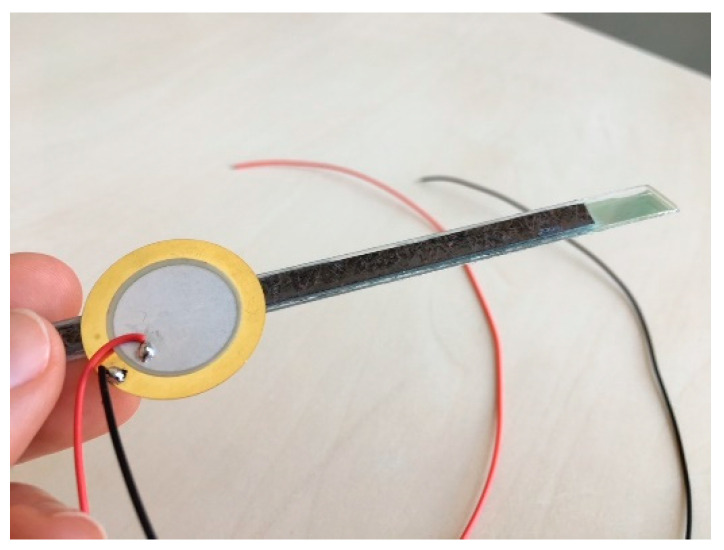
A student-built ME sensor model made out of a strip cut from a CD, a piezo-electric element, and thin steel.

Station 4: Magnetoelectric Sensor. At the last station, students combine the knowledge they have acquired about context, material properties and sensor principles in order to build a functioning ME sensor model from everyday materials. The ME sensor model is based on the basic principle of the ME cantilever beam sensor (Figure 1b), in which a piezoelectric, a magnetostrictive and a substrate material are combined. Strips cut from a CD serve as the substrate material, a piezoelectric element as the piezoelectric material and thin steel (or alternatively the magnetizable metal strip from the inside of security tags) as the magnetostrictive material (Figure 5). The coupling of these materials is achieved by using superglue. The ME sensor model can detect rather large magnetic fields generated by a coil with an AC voltage in the range of U = 10–20 V. This can be used as an opportunity to compare the sensitivity of the ME sensor models built by the students with the ME sensors professionally fabricated in the clean room by the CRC researchers. In the evaluation, building their own ME sensors was considered a highlight by the students. However, it was challenging to adapt the instructional support to the ability of the students. One class felt the sensor design process was too guided when they were given the materials needed for the sensor and were guided through the design process by their supervisor. However, after further opening up the design process by giving different sensor materials to choose from and scaling back support, a third of the student groups failed to develop a working ME sensor (since they chose a magnetostrictive material that was too thick) and were discouraged by the results. The supervisors were instructed to provide step-wise support when needed to resolve this issue.

Conclusion. At the end of the project day, the students compare their approach with the working process of the CRC researchers. This comparison was added to the project day after students noted that they would be interested in learning more about researchers and their daily lives at work. Another element included in the project day for the same reason proved ineffective and is therefore not part of the final design of the project day: We added a media station that provided insights into the work of CRC researchers through various media elements such as a 360-degree video and interviews with CRC researchers. This included, for instance, a description of a typical working day or insights into their personal motivation for their research. The 360-degree video depicted the sensor fabrication in the clean room.

Box 1 **The Magnetostrictive Cantilever Beam Experiment.**

**Figure 6:**
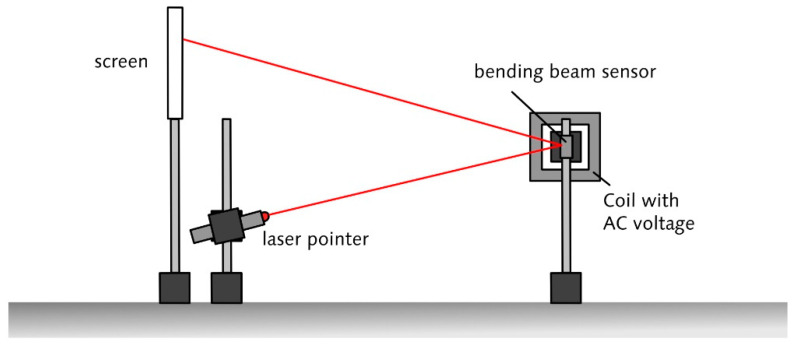
Experimental setup of the bending beam experiment.

This experiment is part of the learning station 3 and illustrates the magnetostrictive effect using a cantilever-type sensor (for more detail see [31]). The magnetostrictive cantilever consists of a non-magnetostrictive substrate material and a magnetostrictive material attached to it. When this cantilever is exposed to a magnetic field parallel to its length, the length of the magnetostrictive material increases while the length of the non-magnetostrictive material remains constant. This causes the cantilever to bend and the tip of the cantilever to be displaced. The displacement is minimal and cannot be seen with the naked eye. We used an optical lever setup to make this effect visible for the students. A laser beam is directed onto the cantilever beam sensor and reflected onto a screen. The experimental setup includes the magnetostrictive cantilever, an electromagnet, a laser pointer and a screen (Figure 6, for more information on the materials see [31]).

The cantilever beam is mounted so that it can be bent horizontally and is inserted inside a coil. A laser beam generated by the laser pointer is directed onto the tip of the reflective cantilever. When AC voltage is applied, the cantilever bends continuously, which in turn leads to the continuous displacement of the laser beam point on the screen. By applying alternating voltage with the self-resonance frequency of the cantilever beam, it is possible to amplify the movement of the cantilever and thus the laser beam point.

This experiment can be integrated and modified in a number of ways for outreach purposes. For its use in the student lab, we focused on students’ autonomous and exploratory learning. Therefore, the experimental setup and a demonstration of a magnetostrictive cantilever that oscillates in self-resonance frequency was presented to the students. Based on the definition of magnetostriction and—if necessary—with the help of prepared tips on cards students were to explain the oscillation of the cantilever. They were then able to use the experimental setup to determine the influence of different design aspects of the cantilever on the performance of the cantilever, while being exposed to different magnetic fields. Factors that could be compared included the thickness of the magnetostrictive material and the respective lengths of the magnetostrictive and non-magnetostrictive materials. These insights prepared the students for the design of their own ME sensor, which was to be manufactured from the materials they got to know in this experiment (with the addition of a piezoelectric material).

Through the design-based research process, first insights about the design of a project day in the context of biomagnetic sensing and the interest of secondary school students regarding research and researchers in the field of biomagnetic sensing could be obtained. The results indicate that the design of the project day might be suitable to motivate the students and foster interest in aspects of biomagnetic sensing and the research of the CRC 1261. It seems that students are especially interested in the work of researchers and aspects of career orientation. Typical questions included: “What does a typical day at work look like? Is there a balance between theoretical and practical work? How do you become a researcher? Should I choose a certain university if I want to become a researcher? What is the best way to decide on a subject to study?” These findings can be used in order to obtain more detailed and robust results concerning the interest of students in researchers and research in the field of biomagnetic sensing in future iterations. 

## 5. Discussion

In this paper, we have shared our ideas for designing an outreach activity, a project day, within the context of biomagnetic sensing and our experiences while developing and implementing it.

Biomagnetic sensing has proven a well-suited topic to inspire outreach activities, even though the content is unusual for classroom learning and is not part of the science curricula. Firstly, it is suitable for integrating STEM disciplines to foster students’ interdisciplinary knowledge and skills. For instance, teaching about piezoelectric and magnetostrictive materials makes it possible to address the structure of matter in more detail, since both phenomena can be explained by the atomic structure of the respective materials. Understanding the structure of matter is an important part of both physics and chemistry education, and knowledge from both disciplines has to be combined to understand the phenomena. In countries with no curricular separation between the natural sciences, it can be stated that learning to connect knowledge within a discipline is as important for successful STEM education as the connection of knowledge across disciplines [1]. In addition, biomagnetic sensing provides a motivating context for school students by connecting the relevance of recent research with a medical problem. Secondly, biomagnetic sensing allows us to integrate aspects about both the nature of science and engineering. By working closely with researchers, it was possible to make authentic references to current research by including depictions of CRC researchers and their work in outreach materials. A facet of science that can be portrayed by the CRC is the facet of enterprising, since within the CRC there are projects that become independent enterprises and products that are introduced onto the market. 

The chosen design approach allowed simultaneous research and design of the project day. The expert consultations we conducted during the design process were fruitful in that they provided new perspectives and led to several improvements before we invited groups of students to test the project day. In particular, the cooperation with CRC researchers proved valuable for our work as it helped to connect outreach activities to authentic research. Not only did the CRC researchers provide feedback on the final prototype of the project day but also contributed to the design of the prototype by, e.g., sharing ideas for model experiments. The feedback methods we used were well suited to resulting in valuable feedback on elements of the project day. We recommend using different evaluation methods such as short surveys, group discussions and interviews, as they provide a holistic insight into participants’ perceptions of the project day. 

There were a number of elements of the project day that were well-liked and received good feedback. For instance, the structure of the project day—embedding the sensor contents into the context of cardiac diagnostics and following the CRC research logic—was comprehensible for school students and seemed to foster their interest in ME sensors. The hands-on experiments were also very well-received. Students stated that they enjoyed exploring the properties of the sensor materials in more details and discovering the function of ME sensors while experimenting. 

Improvements made to the project day included adapting the level of support during the experiments, the connections to CRC research, and the conclusion of the project day. Providing the right level of support during the experiments presented a challenge—with too little instruction, some students were overwhelmed and easily demotivated, with too much instruction, they quickly lost interest. Support staff, such as the learning station supervisors, can resolve this issue by providing adequate support when needed. In addition, the closing of the project day was adapted to the need of the students. During the design process, we learned that the students who tested the project day were interested in the life and work of CRC researchers. To provide insight into these areas, we included a phase in which we compared the development of the model ME sensor during the project day to the actual ME sensor development process in the CRC. To further link the CRC research to the project day, we set up a media station for students to visit during their breaks. Here, they could watch interviews with CRC researchers and a 360-degree video showing sensor development in the clean room during breaks. Since students did not fully utilize the media station, we plan to integrate the media elements in the regular learning stations. 

In the future, the project day will be further evaluated with a larger sample of students, which should lead to further insights into which elements of the project day can particularly foster students’ interest in STEM. Further research on students’ specific interest in biomagnetic sensing will also be conducted to provide an even better foundation for future refinements of the project day as well as related outreach programs. Other plans for the future include establishing boundary activities that connect out-of-school measures such as the project day with in-school learning. Boundary activities increase the likelihood of creating lasting impacts on student interest and learning [33,34] and can take the form of preparation and/ or follow-up lessons at school that address concepts or ideas covered in the project day. Apart from boundary activities, a long-term goal of ours is to connect different outreach formats of the CRC to build a long-lasting, multi-faceted outreach program, making single outreach formats more sustainable.

## 6. Conclusions

In summary, the essential findings for the design of the project day were:

1. The didactic structure with a medical problem as the context and the development of an ME sensor as technological solution offers the students good orientation and has a motivating effect.
2. The experiments provide authentic insights, are easy to use and seem to motivate the students. When conducting the experiments, a balance has to be found between guidance and open inquiry. Therefore, the supervisors should be thoroughly prepared to provide support if necessary. In addition, the tasks should be designed to enable a stronger differentiation according to the performance of the students is possible.
3. The successful execution of the design of the ME sensor is crucial for the students’ sense of competence. Supervisors can help at this stage by providing feedback if necessary. In addition, it is possible to give students more time to troubleshoot and develop a second version of their ME sensor if the first one did not work. In our experience, the problems of a poorly functioning sensor include either the use of the wrong material—an error that is easy to detect when comparing sensors between groups—or insufficient coupling. Insufficient coupling can be detected by closely inspecting the glue layer. In both cases, students are able to correct the errors by designing a second sensor that works if they have enough time and—if necessary—support from other students or their supervisor.
4. A separate media station has not proven effective in giving more insights into the life of scientists of the CRC. In the next design, we will consider integrating media elements that represent aspects of researchers and their work in the CRC directly into the learning stations.

Design-based research is widely described in the literature as a suitable method for designing evidence-based and effective outreach activities. Even though only the first cycles have been realized so far due to the pandemic, we could moderate a fruitful interaction between teachers, educational researchers and researchers in the field of biomagnetic sensing. Teachers were able to make connections to their science curricula, while student questions and feedback pointed out where step-wise guidance was necessary for different groups of students. CRC researchers helped to develop explanations that were appropriate for classroom learning and provided students with authentic scientific insights. We therefore encourage scientists and engineers in the field of magnetoelectric sensing to consider possible outreach activities in their own field and to actively collaborate with educational researchers in co-design processes.

## Supplementary Materials

The following are available online at https://www.mdpi.com/article/10.3390/s21217354/s1.
