# Supplementary material for: Teaching Magnetoelectric Sensing to Secondary School Students—Considerations for Educational STEM Outreach
Source: Sensors (Basel). 2021 Nov 5;21(21):7354. doi: 10.3390/s21217354 (PMC8588487; doi:10.3390/s21217354)
Supplement: Supplementary file 1 [file sensors-21-07354-s001.zip › 360-degree video and interviews.pdf]

Information on science and scientists in the CRC 1261

In order to provide insights into the life of the scientists of the CRC, we used a 360-degree video and interviews with junior scientists at the project day.
The 360-degree video shows a tour through the clean room and can be found here:
https://www.youtube.com/watch?v=J3NIdmMAG98
The interviews can be found at the website of the CRC (http://www.sfb1261.de/index.php/en/mediacenter-en/nachwuchsforschende-wall). Since they were made to reach local students, they are in German. To give an impression of the interviews, we included an interview in English below.

"Working here has changed me" – Interview with Junior Scientists of the CRC

- Nawar Habboush, Digital Communications, M.Sc.
- Laith Hamid, Electrical Engineering, Dr.-Ing.

What are you working on?
Laith: We are working on one application of the sensors investigated at the CRC 1261. Doctors want to localize the brain sources that cause epilepsy, i.e. for correct diagnosis and treatment of epilepsies. It is difficult to interpret indirect measurements on the surface of the body because we measure weaker and mixed versions of the signals and because we also have unwanted signals - from the eye movements, muscle activities, heartbeat and electromagnetic signals from the power supply. We are using source analysis to improve the interpretability of the measurements by solving this so-called "inverse problem"– I am working on the brain and Nawar on the heart.

Nawar: We use a Kalman filter that was especially adapted to the inverse problems of the heart and brain. The Kalman filter is a special algorithm that was also used in 1961 for the moon landing and has often been adapted since then. Ours combines the data with a model about the heart and brain activity developing in time and space so that you can make a prediction.

What is the biggest challenge in your project?
Laith: You observe epilepsy or heart measurements only from the surface so you can never be really sure what is happening inside. The validation of our results is a challenge. We depend a lot on simulations, but also try to use physiological models.

Nawar: That's why we want to combine different measurements, electroencephalography (EEG)-magnetoencephalography (MEG) or electrocardiography (ECG)-magnetocardiography (MCG). The main problem with fusion, however, is that when you have different types of sensors at different locations on the body the tissues will have different sensitivities to the electric and magnetic fields. You need to have the correct position of the sensors and a good model of the different tissues to overcome that.

How does a typical working day look like?

Laith: Most of our work is programming and testing different versions of the Kalman filter – that is quite lonely sometimes (*laughs*). And it needs a lot of patience, but it is rewarding when you see some interesting results. Our version of the Kalman Filter has a good reputation now in our field and we have presented it at several international conferences.

Nawar: My day is often full of technical problems that I need to fix with writing codes and test them. Our office is at the campus of the UKSH but we also go sometimes to the campus of the Faculty of Engineering for meetings with the other groups from the CRC 1261.

What do you enjoy most about your work?
Nawar: When the code works and we get accurate results after hours of developing and testing.

Laith: When you discover something that you have not thought about before and then everything fits together. It is also interesting how enthusiastically the doctors react when they realize they can use our methods to add evidence to their diagnosis and interpret their data.

So, sometimes you surprise each other?
Laith: A lot! At the Faculty of Engineering where I did my master degree most of the people think like me. However, the interdisciplinary work in the CRC has changed me. I use a different style of presentation now. I translate between technology and medicine, like an interface.

Nawar: The doctors look at the problems from a different perspective and make really helpful suggestions without knowing all the technical details. They see different things than we do.

Why did you choose this research field?
Nawar: I was going into the field of signal processing and could choose between speech recognition and a medical application. I thought that it was more valuable for me to do medical research.

Laith: Personally, I lost some close people due to epilepsy. If I can contribute even a small thing towards a better understanding or even a method to diagnose or treat epilepsies, I will do that.

How do you explain what you are doing at a family event?
Laith: We are trying to find the sources that cause epileptic seizures. With source localization we are working in a small corner of algorithmic research, nobody is competing with each other here.

Nawar: I say, I am working on the analysis of magnetic fields from the heart and measure the signal on the surface to find the sources inside the heart to separate normal from abnormal

heart activity. This can be useful for the doctors because nowadays they use surgery with invasive cardiac catheters but performing the diagnosis from the outside is better for the patients. My family often reacts impressed, because they did not know there is research in this field.

What kind of feeling is that, to be where nobody else has been?
Laith: On the one hand, it is easier, because your papers will not be compared with a lot of others. On the other hand it feels like being in a dark forest where you have to find your own way by yourself. It can be frustrating because sometimes you reach a dead end and have to find another way.

What do you do then?
Nawar: I leave my desk for a pizza and think about something else for a while, then come back to my desk when I have a relaxed mind and try to look at the problem from another angle.

You both studied the international master program "Digital Communications" at the CAU – why would you say it was perfect for you?
Laith: You can get a taste of lots of topics and then choose your future specialization. I came as a DAAD scholarship holder from Iraq and did my bachelor's degree at the University of Baghdad. Kiel is a nice city by the sea with amazing nature.

Nawar: I also came as a DAAD scholarship holder from Iraq, where I studied Communications Engineering. I had a few admissions from universities but here the diversity of the master program was the best.
