# Supplementary material for: Teaching Magnetoelectric Sensing to Secondary School Students—Considerations for Educational STEM Outreach
Source: Sensors (Basel). 2021 Nov 5;21(21):7354. doi: 10.3390/s21217354 (PMC8588487; doi:10.3390/s21217354)
Supplement: Supplementary file 1 [file sensors-21-07354-s001.zip › Station 1/Diagnostic guide and description of possible findings.pdf]

Guide for the interpretation of an ECG curve

On the basis of an ECG, many characteristics of the heart can be detected and malfunctions can be uncovered. To evaluate an ECG, it is common to go through a series of analysis steps.

We go through a small part of them, which enables us to differentiate between certain malfunctions and consequently to make a diagnosis. The analysis steps we go through enable us to examine the basic rhythm of the heart. The basic rhythm can be analysed on the basis of two properties, the relationship of the P wave to the QRS complex and the regularity of the cardiac action.

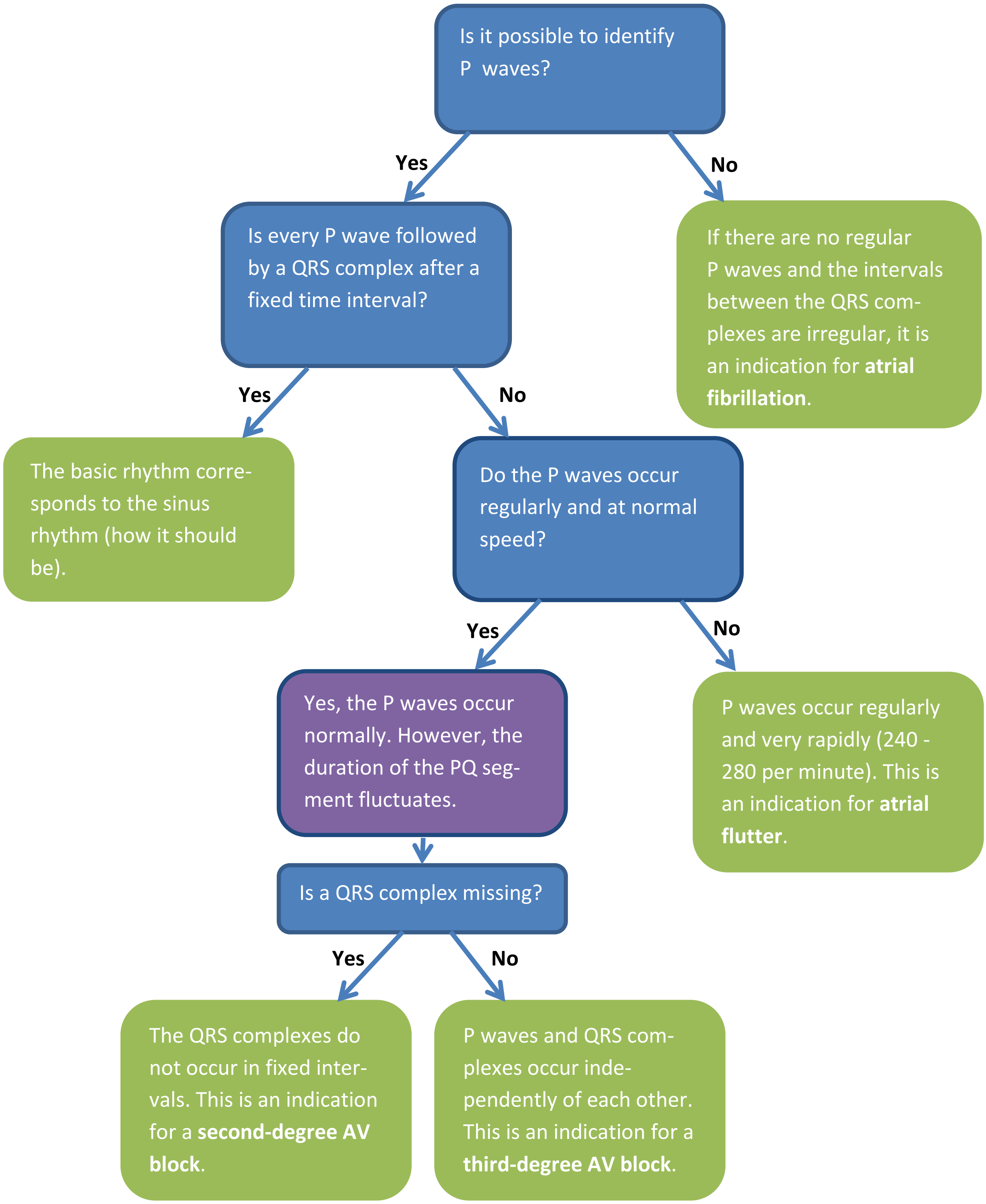

More Information on the Diagnoses

Normal findings

The heart beats normally, the basic rhythm corresponds to the sinus rhythm.

There are no abnormalities of the transmission of electrical excitation apparent.

Atrial fibrillation

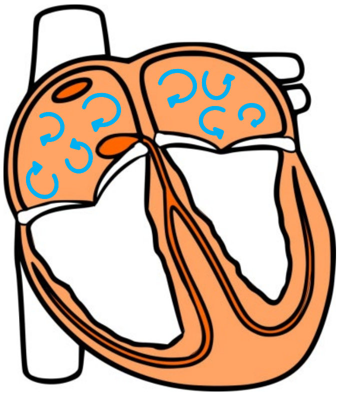

Atrial fibrillation refers to the condition in which the electrical impulses in the atria travel uncontrollably in all directions. Due to damaged heart muscle cell tissue, impulses can no longer be transmitted to the AV node in a straight line. This results in electrical impulses that permanently form small circles in the atria. The AV node acts as a filter for these rapidly successive impulses, as it transmits the electrical impulse to the ventricles at a slower rate. As a result, only a fraction of the excitation impulses reach the ventricles, which usually contract at a normal rate, albeit irregularly.

Atrial flutter

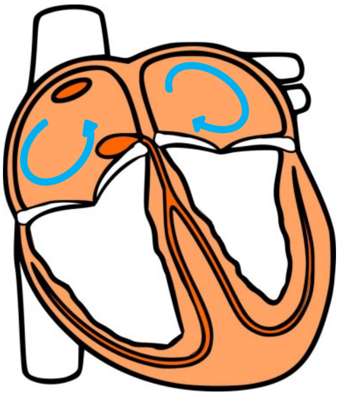

Atrial flutter refers to the condition in which excitation circulates rapidly and regularly in the atria, causing them to contract at a rate of up to 300 beats per minute. Since the AV node transmits the excitation at a slower rate, it acts as a filter that transmits only a fraction of the excitation impulses to the ventricles. As a result, the ventricles usually contract at a relatively normal frequency.

Third-degree AV Block

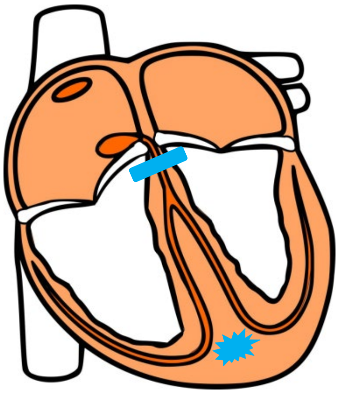

Atrioventricular block (AV block for short), is a term for a disturbance in the transmission of excitation in the AV node or in the subsequent excitation transmission system. Depending on the severity of the block, AV blocks are classified into different degrees. The most severe form is the third-degree AV block, in which conduction between the atria and the ventricles is completely interrupted. In some cases, an electrical impulse is generated in the ventricles: a replacement rhythm that runs independently of the sinus rhythm. At worst, no replacement rhythm is generated, the ventricles do not contract, and no more blood is pumped out of the ventricles. The patient dies.
