# Supplementary material for: Teaching Magnetoelectric Sensing to Secondary School Students—Considerations for Educational STEM Outreach
Source: Sensors (Basel). 2021 Nov 5;21(21):7354. doi: 10.3390/s21217354 (PMC8588487; doi:10.3390/s21217354)
Supplement: Supplementary file 1 [file sensors-21-07354-s001.zip › Station 1/Information text - transmission of excitation.pdf]

Electrical signals of the heart

Electrocardiography measures electrical signals of the heart, allowing conclusions to be drawn about cardiac activity. But why do electrical signals exist in the heart?

Muscle contraction and electricity are related: When muscle cells are electrically excited, they contract (this is why people who stick their finger in an electrical socket twitch all over their bodies - the current flows through their muscles, which contract). Of course, the same is true for heart muscle cells. In order for the individual heart muscles to contract, the heart muscle cells must be electrically excited. In this process, an electrical impulse travels through the heart.

In order for the heart to beat normally, i.e. to pump enough blood through the veins at an appropriate rate, the electrical impulse must travel through the heart along a certain path. An electrocardiogram (ECG) makes it possible to visualize this transmission of electrical excitation within the heart, which allows to draw conclusions about possible heart diseases. In order to be able to interpret the ECG later on, we need to understand the transmission of electrical impulses in the heart.

The electrical excitation of the heart originates in the so-called sinus node, the heart's “clock generator”. The sinus node determines how often the heart beats per minute (which is about 60 to 70 times if you are healthy and at rest). The sinus node consists of specialized heart muscle cells, the pacemaker cells, and is located in the wall of the right atrium. From the sinus node, an electrical impulse travels through the atria to the AV node (atrioventricular node), the "gatekeeper" between the atria and the ventricles. Because of the electrical impulse, the atria contract and pump blood into the ventricles. Meanwhile, the AV node gathers the electrical excitation from the atria and slowly transmits it to the ventricles. There, the electrical excitation spreads through conduction pathways ("highways" for electrical excitation) to the apex of the heart, from where it spreads to the rest of the ventricles. Due to this, the ventricles contract and pump blood into the arteries.

How can we make this transmission of electrical excitation visible? For this, it is important to know how the electrical excitation is transmitted within the human body. Transmission of excitation takes place at the cellular level. Non-excited cells are negatively charged on the inside and positively charged on the outside. If they are excited, the charge ratio is reversed and they are now positively charged on the inside and negatively charged on the outside. After excitation, this charge reversal is slowly reversed until the original charge state of the cell is restored.

We can now combine the knowledge of the paths on which the electrical impulse travels through the heart and the charge changes of electrically excited cells: In figure 1 we can see an excitation and thus an electric dipole travelling through the heart. This traveling dipole generates an electric field, which can be measured with the help of the ECG sensors.

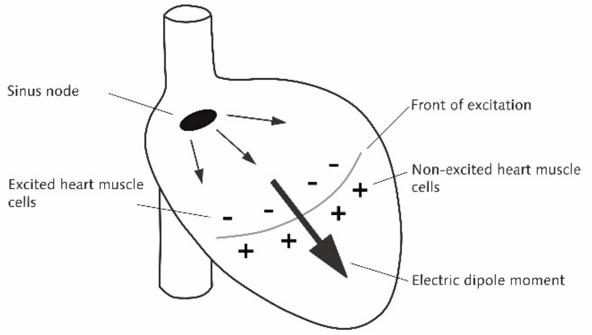

Figure S1. The human heart as time-varying electrical dipole.
