# Supplementary material for: Teaching Magnetoelectric Sensing to Secondary School Students—Considerations for Educational STEM Outreach
Source: Sensors (Basel). 2021 Nov 5;21(21):7354. doi: 10.3390/s21217354 (PMC8588487; doi:10.3390/s21217354)
Supplement: Supplementary file 1 [file sensors-21-07354-s001.zip › Station 1/Instructions for supervisors_station1.pdf]

Station 1 – Electro- and Magnetocardiography

Brief Overview:
At this station, students are able to deepen their knowledge of the transmission of electrical impulses in the heart and transfer this knowledge to the operating principle of electrocardiography. Students will produce an electrocardiogram (ECG for short) and assign the corresponding excitation states of the heart to the individual areas in the ECG. Later on, students will interpret two ECGs, one of a normal, healthy heart, the other of a heart with a typical illness. Electro- and magnetocardiography are then compared and the respective advantages and disadvantages are discussed.

Duration: 60 minutes

Participants: 3 - 5 students (one group)

Time table:

Time	Topic	Short description
20 min	Theoretical introduction	Learning about the structure of the heart and about the operating principle of electrocardiography
15 min	General information on recording and interpreting an ECG	Producing an ECG<br>Learning about a typical ECG curve and connecting its form to the structure and functioning of the heart
15 min	Making a diagnosis for two example cases	Using a diagnosis guide for interpreting ECG curves and explaining the results
10 min	Comparison of magneto- and electrocardiography	Comparing electro- and magnetocardiography and discussing the respective advantages and disadvantages

Some of the content to be worked on may already be known to the students. Thus, at the beginning of the station, supervisors should find out what prior knowledge students have. Students should have the opportunity to introduce their knowledge on the topic to the discussion and – if able – to explain the transmission of electrical excitation. In this case, the supervising person should only support by providing additions or illustrations.

Theoretical Introduction (20 min)

This station ties in directly with the introduction of the project day. In the introduction, the importance of medical-technical innovations for the diagnosis and treatment of cardiovascular diseases was emphasized. In addition, a magnetoelectric sensor that is currently being developed by a collaborative research center (CRC 1261) at the University of Kiel, has been talked about. It has been said, that this sensor can be used for cardiac diagnostics by recording a magnetocardiogram (MCG for short). In addition, the functioning of the heart and an ECG measurement was roughly described. The introduction to this first learning station should revive the introduction to the project day by asking what exactly it is that an ECG measurement measures.

An ECG measurement measures electrical potential differences. But when and why do electrical processes take place in the heart? Here, the students have the opportunity to express their knowledge/assumptions. They are then given an information text about the transmission of electrical

excitation in the heart. After they have read the text, they can ask questions and are then to summarize the text.

The content of the text is followed up by asking the students what they know about electric fields. Answers are collected and discussed. At the end, the form of electric fields should have been discussed as well as their propagation (they can only propagate well in conductive media). Subsequently, these findings can be applied to the context of the heart:

The electric field created by the time-varying dipole – the heart – is still measurable on the surface of the body because the heart is embedded in a conducting medium. As a result, by measuring the electric potential of different points on the body surface, it is possible to draw conclusions about the electrical activity of the heart. Each point of the electric field measurable on the body surface has a certain potential. Points of the same potential are represented on so-called equipotential lines. The potential of the body surface can be recorded by ECG electrodes (Figure 1). From these recordings, conclusions can be drawn about cardiac activity. This means that ECGs can be used to diagnose cardiac arrhythmias and heart attacks, among other things.

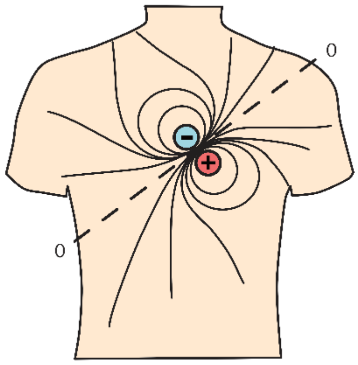

Figure S1. Equipotential lines of the electrical potential of the human heart.

After talking about these basics for cardiac diagnostics, students will learn about the typical form of an ECG curve and how to draw simple conclusions from this curve. For this, they are to create a typical ECG curve by arranging parts of the curve in the right order. A self-recorded ECG curve of a participant can serve as an orientation for this endeavour.

General Information on Recording and Interpreting an ECG (15 min)

For recording an ECG, students use a single-channel interpretive electrocardiograph (*Cobra SMARTsense* by *Phywe: https://www.phywe.com/equipment-accessories/measurement-devices/physiology-measuring-instruments/cobra-smartsense-ekg-0-4-5-mv-bluetooth-usb_2037_2968/*). This electrocardiograph is developed for didactical purposes only and is therefore not to be used for clinical diagnoses.

Single-channel interpretive electrocardiographs are used for patient monitoring and long-term examinations and can visualize arrhythmias and ventricular fibrillation. However, they are not suitable for further diagnoses, for which more electrodes would be needed.

For recording the ECG, several things have to be kept in mind: the electrodes have to be firmly attached at the right positions, electrical devices like smartphones should not be close to the body and the “patient” should lie or sit without moving. Students can look at the recorded ECG via an app on a smartphone or tablet.

An ECG curve of a healthy heart has a typical shape (Figure 2). The group receives a schematic ECG curve that has been cut apart and has to arrange the parts in the right order. The self-recorded ECG can serve as an aid. After the curve is complete, the students repeat their knowledge about the transmission of electrical impulses in the heart and try to transfer this to the ECG curve. To do this, they are told that a cardiac cycle extends from the P wave to the T wave. They can of course be given tips and have questions answered during this task. To help them, they are given pictures of the individual cardiac excitation states, which they can assign to the ECG curve (Figure 3).

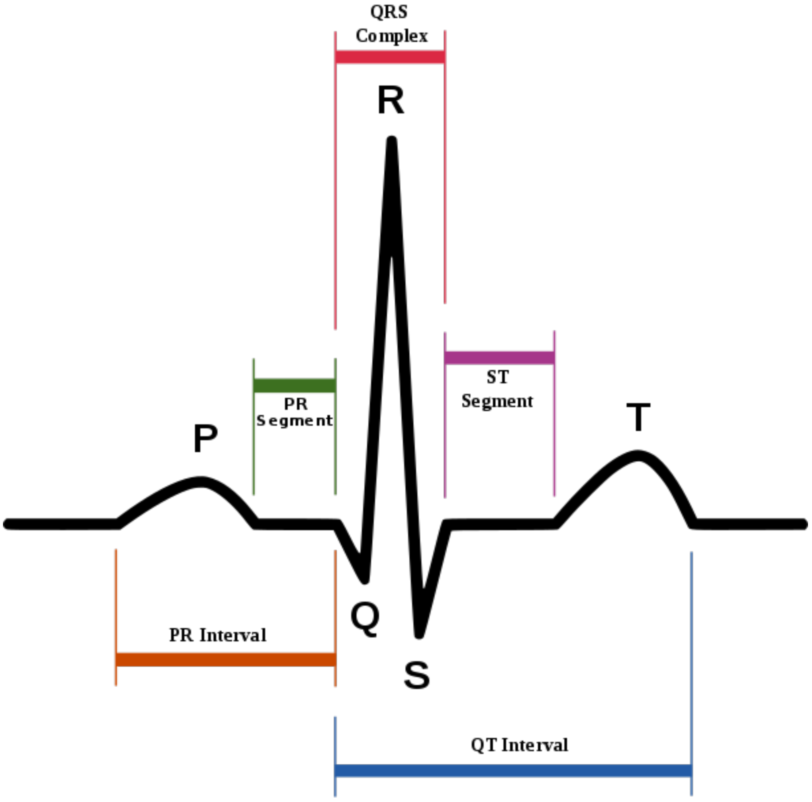

Figure S2. Schematic ECG curve.

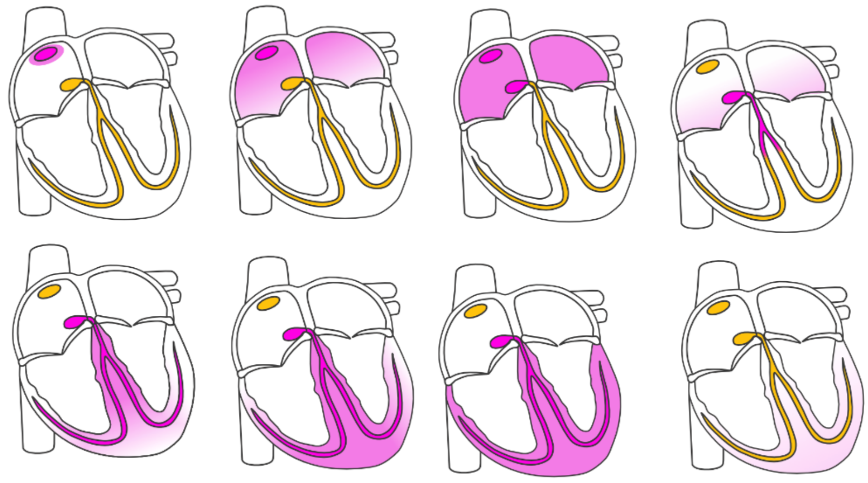

Figure S3. Cardiac excitatiation states.

In the end, the students should have obtained the following result: The excitation of the sinus node is not visible because too few cells are involved. The excitation propagation from the sinus node through the atria is visible as a P wave. The excitation propagation at the AV node is not visible because too few cells are involved, we see this phase as PR segment. Excitation propagation through the conduction pathways, the "highways" for electrical excitation, is again not visible because too few cells are involved. The propagation of excitation from the conduction pathways to the ventricles is visible in the QRS complex. This is where most of the heart muscle cells are involved, so it is visible as a large peak. When all the cells of the ventricles are excited, this is depicted by the ST wave. The T-wave signals the reduction of excitation in the ventricles.

To document this, students work on task 1 of the worksheet. This task is at the back of the worksheet as it shows an ECG curve that students previously had to construct themselves.

Making a Diagnosis for Two Example Cases (15 min)

In the following, students receive two ECG curves to interpret using a diagnostic guide. One of the curves belongs to a normal, healthy heart, the other shows a heart with a typical cardiac illness (atrial fibrillation, atrial flutter and third-degree AV block). The students work on this task among themselves; if the group is large (five students), it can be considered to divide them into two teams who start with different ECG curves to increase the activity of each. In addition to the diagnostic guide and the two ECG curves, students also receive brief descriptions of possible findings. With the help of these descriptions, students can explain their diagnoses by explaining why the ECG signals look the way they do.

The interpretations of the ECG signals are then discussed. In particular, it is discussed why it is logical to see the respective ECG curve in the case of a certain heart disease.

Comparison of Magneto- and Electrocardiography (10 min)

At this point, the students have dealt with the ECG measurement quite a bit. They are now asked to spontaneously name the advantages and disadvantages of this measurement in terms of the comfort of the measurement, the accuracy, the costs and mobility (here, of course, they can only talk about their personal view!).

Based on this, they are asked what they remember about the MKG measurement, which was briefly discussed at the introduction of the project day, and how this is related to the ECG. The connection with the ECG is based in physics: every electric field generates a magnetic field. This means that instead of measuring the electric field of the heart, the magnetic field generated by the electric field can be measured as well. Magnetocardiography, like electrocardiography, is thus based on the measurement of the changing dipole of the heart.

But why should this measurement have advantages over ECG measurement? And are there also disadvantages of this form of measurement? The students receive a flyer about magnetocardiography and read it in order to find advantages and disadvantages of magnetocardiography.

One advantage of magnetic fields in contrast to electric fields is that they are not shielded or influenced by biological materials (bones, tissue, etc.). Thus, the magnetic measurement is not distorted like the electric measurement and non-contact measurements are possible. This leads to increased accuracy and comfort during the measurement. However, since the biomagnetic fields are very weak, they are easily superimposed by other magnetic fields (such as the earth's magnetic field).

That is why they must be carried out in shielding chambers. In addition, the magnetic field sensor that is currently used in medicine needs to be cooled down to about -269 °C.

The students should now fill in the table in task 2, in which ECG and MKG are compared.

Regarding	Better/ worse than the ECG	Explanation
Costs	Worse	High costs due to constant cooling of the sensor and the purchase of the shielding chamber
Mobility	Worse	Not suitable for mobile use because of the shielding chamber and the cooling installation of the sensor
Precision of measurement	Better	High accuracy, since signals are not influenced by surrounding body substances
Convenience	Better	Non-contact measurements are possible
Comparability of measurements performed at different times	Better	The measurement is not dependent on the positioning of the electrodes

After filling out the table, they should name possible improvements for the current MKG measurement (the two major ones are: no need for the shielding chamber and a sensor that works at room temperature).

Reserve: If there is time left, the students can think about why MKG measurements can be helpful in the following cases: severely overweight patient; emergency with acutely injured person; examination of cardiac arrhythmias in unborn children.
