# Supplementary material for: Teaching Magnetoelectric Sensing to Secondary School Students—Considerations for Educational STEM Outreach
Source: Sensors (Basel). 2021 Nov 5;21(21):7354. doi: 10.3390/s21217354 (PMC8588487; doi:10.3390/s21217354)
Supplement: Supplementary file 1 [file sensors-21-07354-s001.zip › Station 1/Worksheet_station1.pdf]

1. *Structure of the Human Heart*

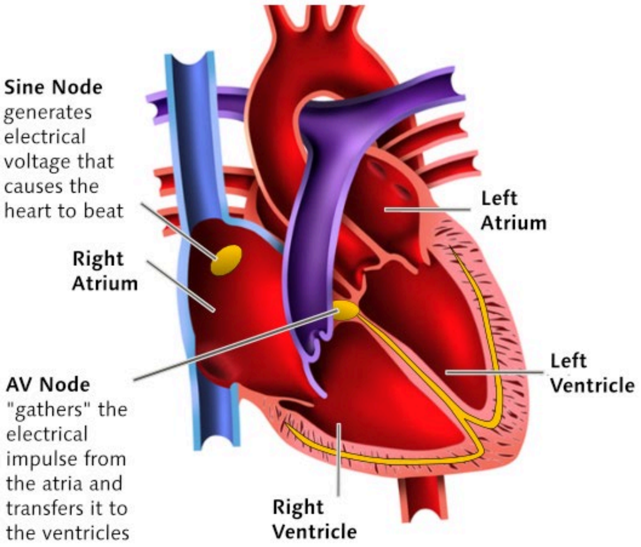

Figure S1: Schematic diagram of the human heart.

3. *Comparing Magnetocardiography and Electrocardiography*

Like the electrocardiogram, the magnetocardiogram (MCG) measures the change in electrical potential generated by the heart. While the ECG measures the heart's electric field, the MKG measures the heart's magnetic field. This type of measurement is already used in clinical diagnosis.

Task 2

Note in which points the MCG measurement is better or worse than the ECG measurement. Explain your decision briefly.

**Regarding**	**Better/ worse than the ECG**	**Explanation**
Costs		
Mobility		
Precision of measurement		
Convenience		
Comparability of measurements performed at different times		

2. *The Electrocardiogramm (ECG)*

Task 1

An electrocardiogram (ECG for short) can be divided into different sections, each of which depicts a different phase of a heart beat (Figure 2). Figure 3 shows different stages of excitation of the heart, however they are in the wrong order.

Put the images of the heart in the correct order and match them to the appropriate sections of the ECG. Write down your results by entering the corresponding number and letter combination in the field above the respective heart.

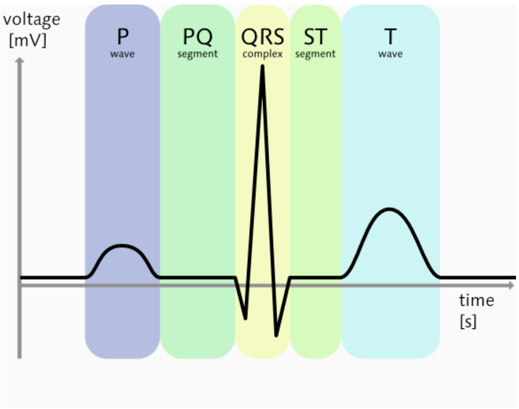

Figure S2: Schematic diagram of an ECG of a nor-mal, healthy heart.

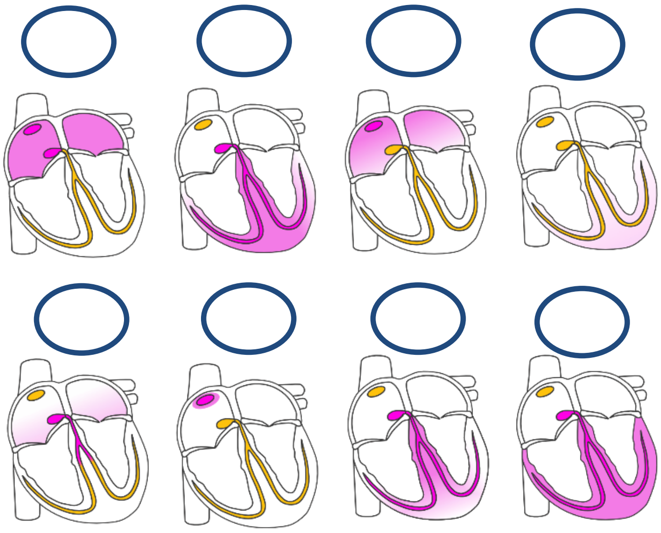

Figure S3: Different stages of electrical excitation of the human heart.
