# Supplementary material for: Teaching Magnetoelectric Sensing to Secondary School Students—Considerations for Educational STEM Outreach
Source: Sensors (Basel). 2021 Nov 5;21(21):7354. doi: 10.3390/s21217354 (PMC8588487; doi:10.3390/s21217354)
Supplement: Supplementary file 1 [file sensors-21-07354-s001.zip › Station 2/Instructions for supervisors_station2.pdf]

## Station 2 – Piezoelectricity

**Brief Overview:**
At this station, students will learn about the phenomenon of piezoelectricity. First, the phenomenology is explained, then a model-based explanation is provided, and finally a demonstration experiment is performed and interpreted.

**Duration:** 45 minuten

**Participants:** 3 - 5 students (one group)

**Time table:**

| Time | Topic | Short description |
|---|---|---|
| 20 min | Theoretical introduction | Learning about the phenomenon of piezoelectricity, piezoelectricity in everyday life and a model-based explanation of piezoelectricity |
| 17 min | Experiment | Conducting the experiment and – if wanted – comparing the results with other groups |
| 8 min | Analysis | Documenting and evaluating the experiment |

**Theoretical Introduction (20 min)**

The phenomenon of piezoelectricity is not a mandatory topic at school. Thus, it can be assumed that the students are hearing about it for the first time.

At the beginning, the students are informed that at this station they have the opportunity to learn about important properties of a material that they will also use in their own sensor construction. Therefore, they should keep in mind the following question: "What properties of piezoelectric materials are important for sensor construction?"

Examples from the students' everyday lives can be used as an introduction to piezoelectricity. For example, they can be asked about how an airbag or a lighter works. In both cases, a piezoelectric substance plays a key role. This substance is deformed by a mechanical force and thus builds up an electrical voltage which, in the case of the lighter, is discharged as a spark, igniting the gas. Another example of an application of piezoelectricity may sound more futuristic: In New York as well as in Las Vegas, sidewalks with integrated piezoelectric sidewalk panels are being tested, which convert the mechanical energy of pedestrians' steps into electrical energy for lighting street lamps.

Then, the term piezoelectricity is defined. Certain substances are piezoelectric. These substances are electrically neutral in their resting state and generate an electrical voltage when mechanically deformed. Depending on the geometry of the substance, this generated voltage is parallel, perpendicular or diagonal to the exerting force. The students write down the definition of piezoelectricity in their own words.

Piezoelectricity can be explained using a model. For this, students are asked to share their chemical knowledge of the structure of salts or salt crystals. Salts consist of positively and negatively charged particles – cations and anions. When salts crystallize, salt crystals are formed in which the ions are arranged regularly. Here, the ions in an electrically neutral substance are arranged in a way that the negative charge concentration $Q^-$ and the positive charge concentration $Q^+$ coincide (Figure 1). Due

to this, no electrical voltage occurs. By deforming the substance, the charge concentrations shift and generate an electric voltage, which we call piezoelectricity (Figure 2).

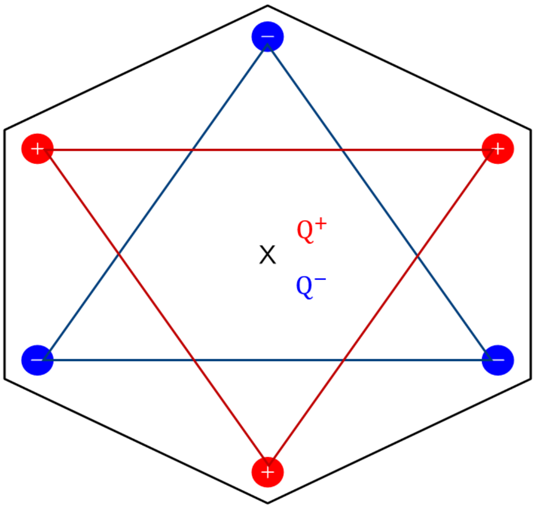


**Figure S1**. Piezoelectric substance in its resting state – the charge concentrations coincide.

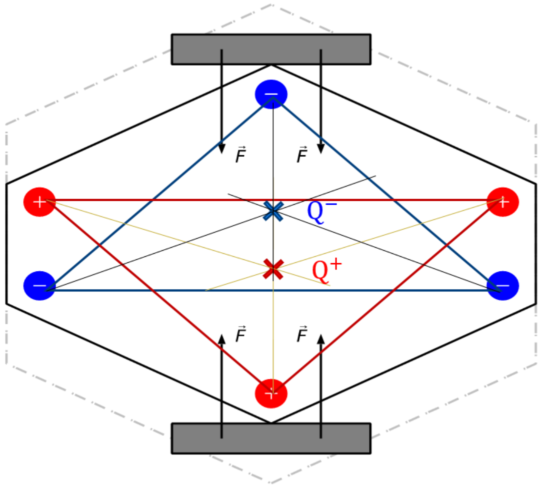


**Figure S2.** Piezoelectric substance when a force is applied – the charge concentrations shift and an electric volt-age is generated.

To see this, students complete task 1a on their worksheet, in which they construct the newly created charge concentrations. For this, they need to know how to construct the centroid (also called center of mass) of a triangle: It is the the point where the three medians meet (Figure 2). After this, they work on task 1b.

Then they are asked if they can imagine what the "inverse piezoelectric effect" might be. After the students describe their ideas about it, the correct answer is repeated or given: Piezoelectric substances deform when a voltage is applied. Students write down this answer in task 2.

**Experiment (17 min)**

Now, the students receive a piezoelectric crystal, or more precisely a Rochelle salt crystal, with which they are to generate and measure as much voltage as possible.

For this purpose, a short introduction is given about the structure of crystals and the growing of crystals. Crystals are piezoelectric if they do not have a center of symmetry. The chemical formula of Rochelle salt is presented and the different ionic radii of sodium ions and potassium ions are addressed (Figure 3). As a result of the different ionic radii, Seignette salt is asymmetric and crystallizes into a crystal without a center of symmetry, forming a piezoelectric substance. This explanation can be adapted depending on the chemistry knowledge of the group - classes that did not have chemistry for two or three years do not need to be confronted with the structural formula of Rochelle salt.

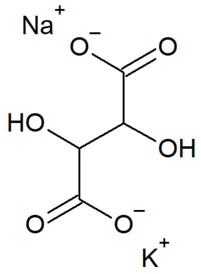

Abb. 3: Rochelle salt (potassium sodium tartrate).

After talking about this basic knowledge, the students are now encouraged to measure as much voltage as possible while mechanically deforming the crystal. Doing so, they must be careful not to exert too much mechanical pressure on the crystal, as this could cause it to break. For measuring the voltage, they have to combine the materials offered in a reasonable way so that generating and measuring voltage are possible.

*Hints*: When a static force is applied, no voltage can be measured because the voltage is quickly dissipated by current flow. Thus, the voltage can only be measured directly when the mechanical force is first applied. One way to generate voltage is to fix the crystal, attach aluminum foil to two opposite sides (which serve as electrodes) and connect the electrodes to a multimeter using alligator clips. It has to be ensured that the aluminum foil is not in contact with electrically conductive objects. To prevent this, insulating material (foam rubber, modelling clay etc.) can be used. Then a non-conductive object (for example a pencil) can be used to tap lightly on the crystal. If done correctly, voltage should be measured by the multimeter.

Students can then compare different crystals based on the maximum voltage they can generate. Are there differences if the crystals are of different size or regularity? The regularity of the crystals should be the main factor, the most regular crystals generating the highest voltage.

**Analysis (8 min)**

Students complete the sentence on their worksheet, recording the core observation (task 3): the higher the mechanical force on the piezoelectric substance, the greater the measured voltage.

The supervisor then shows a piezoelectric element that the students will use when building their sensor. Here, a piezoelectric substance is applied to a brass plate. The wires of the piezoelectric element can be connected to a multimeter using alligator clips and light pressure applied to the crystal with the fingers. High voltage values should be detectable. It can be discussed why these amounts are much higher than those observed in the home-grown crystals.

As a conclusion, the students receive a card:

**Piezoelectricity**

**electrical ↔ mechanical**
