# Supplementary material for: Teaching Magnetoelectric Sensing to Secondary School Students—Considerations for Educational STEM Outreach
Source: Sensors (Basel). 2021 Nov 5;21(21):7354. doi: 10.3390/s21217354 (PMC8588487; doi:10.3390/s21217354)
Supplement: Supplementary file 1 [file sensors-21-07354-s001.zip › Station 2/Worksheet_station 2.pdf]

1. *Before the Experiment*

Definition of **piezoelectricity:**____
____
____

Task 1

a) Complete Figure 2 by constructing the charge concentrations Q^+ and Q^- of the three positive and the three negative charge concentrations respectively.

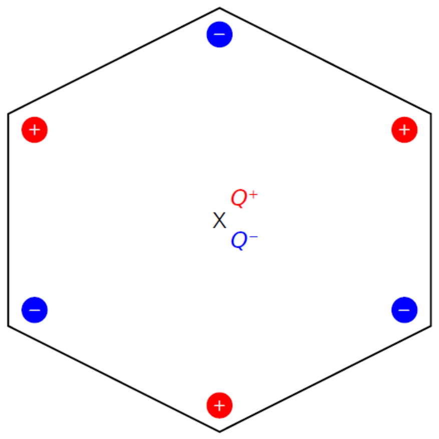

Figure S1. Piezoelectric substance without a force being applied.

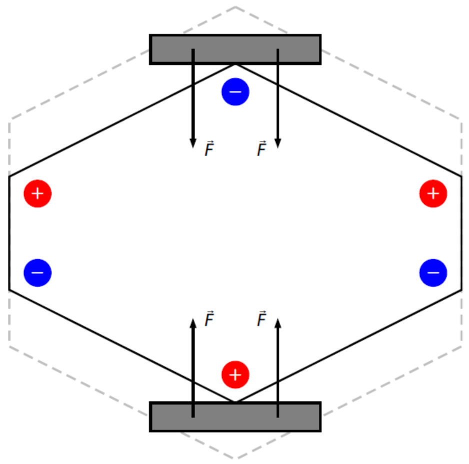

Figure S2. Piezoelectric substance when a force $\vec{F}$ is applied.

b) In which direction the voltage is formed in Figure 2 – perpendicular, parallel or diagonal to the applied force?

____

Task 2

In addition to the piezoelectric effect, there is a so-called *inverse piezoelectric effect.* Describe it in your own words.

____
____
____

2. *After the Experiment*

Task 3

Summarize your observations by noting the relationship between the force exerted and the voltage measured in the following sentence:

The higher the mechanical force on the piezoelectric substance, the ___________________________
____.
