# Supplementary material for: Teaching Magnetoelectric Sensing to Secondary School Students—Considerations for Educational STEM Outreach
Source: Sensors (Basel). 2021 Nov 5;21(21):7354. doi: 10.3390/s21217354 (PMC8588487; doi:10.3390/s21217354)
Supplement: Supplementary file 1 [file sensors-21-07354-s001.zip › Station 3/Cards with hints and questions.pdf]

Cards with additional information for explaining the experiment

Electromagnetism

Electric and magnetic fields are interrelated - every electric field generates a magnetic field. A current I flowing linearly (for example through a cable) generates a magnetic field $\vec{B}$ whose field lines form circles around the current-carrying conductor. A current-carrying coil also generates a magnetic field. In the center of the coil, the magnetic field lines run almost parallel; a north pole (N) is formed on one side of the coil and a south pole (S) on the other side.

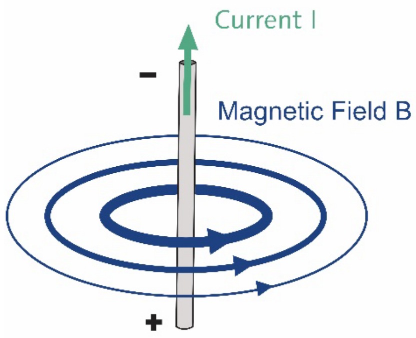

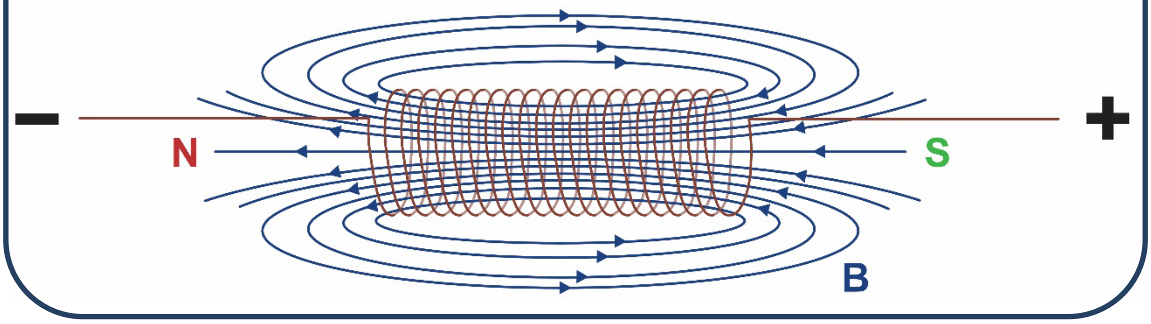

Magnetostriction

Magnetostrictive materials change their shape and/or size when they are exposed to a magnetic field. The magnetostrictive material expands in the direction of the applied magnetic field.

Resonance Frequency

Every object has a so-called resonance frequency. When oscillating an object at its resonance frequency, the oscillation is amplified. If, for example, a glass is made to oscillate/ vibrate at its resonant frequency over a longer period of time, the vibrations become stronger and stronger and the glass may break. The specific resonance frequency of an object is determined, among other things, by the mass, shape and pliability of the material.

Alternating Current (AC)

In an electric circuit, the electric current flows from the positive to the negative pole. In the case of alternating current, the polarity changes periodically, i.e., the positive and negative poles alternate at a fixed frequency. This frequency is the frequency of the alternating current.

Cards with hints and questions for the explanation of the experiment

The structure of the sensor is crucial: What materials does the sensor consist of? How do the individual materials change in the presence of a magnetic field?

How are the magnetic field of the coil and the AC voltage related?

In the experiment, signals are converted. Which signal is the starting point? Trace the path of this signal - into which other signals is it converted?

What is the role of the laser pointer?

Observe the laser stripe on the screen through a slow motion camera.

Research Questions for Further Work with the Experiment

1) Determine in which direction the bending beam sensor is deflected by using the laser pointer.

2) How does the strength of the magnetostriction change with increasing (and decreasing) current strength?

3) What effect does the width of the magnetostrictive material have on the behavior of the sensor?

4) Which properties of the magnetostrictive material could also influence the sensor behavior?
