# Supplementary material for: Teaching Magnetoelectric Sensing to Secondary School Students—Considerations for Educational STEM Outreach
Source: Sensors (Basel). 2021 Nov 5;21(21):7354. doi: 10.3390/s21217354 (PMC8588487; doi:10.3390/s21217354)
Supplement: Supplementary file 1 [file sensors-21-07354-s001.zip › Station 3/Instructions for supervisor_station3.pdf]

Station 3 – Magnetostriction

Brief Overview:
Bei dieser Station sollen Schülerinnen und Schüler das Phänomen der Magnetostriktion kennenlernen. Hierfür wird zunächst die Phänomenologie erläutert und im Anschluss ein Modellversuch vorgeführt. Die Schülerinnen und Schüler analysieren und erklären den Aufbau dieses Modellversuchs. Nachdem sie den Aufbau verstanden haben und erklären konnten, gehen sie mithilfe des Modellexperimentes Fragen nach, die für die Konstruktion eines eigenen Sensors von Bedeutung sind.

Duration: 45 minutes

Participants: 3 - 5 school students (one group)

Time table:

Time	Topic	Short description
5 min	Short introduction	Learning about the phenomenon of magnetostriction<br>Demonstrating the experiment
15 min	Explaining the Experiment	Analysing the experiment<br>Explaining the experiment
20 min	Conducting research with help of the experiment	Answering questions with help of the experiment<br>Recording of results

Short Introduction (5 min)

The phenomenon of magnetostriction is not a mandatory topic at school. Thus, it can be assumed that the students are hearing about it for the first time.

At the beginning, the students are informed that at this station they have the opportunity to learn about important properties of a material that they will also use in their own sensor construction. Therefore, they should keep in mind the following question: "What properties of magnetostrictive materials are important for sensor construction?"

As an introduction to the topic, students are told about magnetostriction in their everyday life: the humming of transformer stations, certain ceiling lamps and older computers originates in magnetostrictive materials. The iron cores of the transformers are magnetostrictive and therefore expand along the magnetic field, which is generated by the electric field. As this field permanently changes orientation due to the alternating current, the iron cores change length at the corresponding frequency. The AC voltage in the power grid has a frequency of 50 Hz, so the iron core changes its length $50 \cdot 2 = 100$ times per minute. The resulting vibrations cause loose mechanical parts in the devices to oscillate, which we perceive as a hum.

Based on this introduction, the supervisor can define the term “magnetostriction” which students can document on their worksheets.

Nach dieser Einleitung wird der Begriff „Magnetostriktion“ definiert. Die betreuende Person gibt hierbei die Definition vor und die Lernenden notieren sie sich auf ihrem Arbeitsbogen. Magnetostrictive materials are substances that change their size and/or shape as soon as they are exposed to a magnetic field. Since this effect is very small (only 0.01 to 0.001% of the material expands, so only

0.1 mm or 0.01 mm per meter of extension can be seen), various "tricks" can be used to make this effect visible to the naked eye.

In the following, the learners are presented with a model experiment in which magnetic signals are converted into optical signals. This is achieved by using magnetostrictive materials and some of the "tricks" mentioned earlier.

The experimental setup is based on the bending beam principle. This principle can be observed if two materials with different elongation behavior are attached to each other. A longitudinal expansion of one material (in our case the magnetostrictive material) causes a deflection of the sensor in the direction of the material that does not expand at all or expands to a lesser extent. In the case of our experimental setup, the magentostrictive material (thin steel) is attached to a carrier layer (a CD strip).

The experiment is based on the fact that the bending beam sensor is excited to oscillate by the magnetic field of a coil. The oscillation frequency is determined by the frequency of the alternating voltage, which is applied with the aid of a function generator. By adjusting the frequency, the bending beam sensor can be made to oscillate at its resonance frequency, which causes the oscillation to amplify. This amplified oscillation can be visible to the naked eye as a vibration of the sensor. The laser pointer is used to better visualize the vibration of the sensor: It is directed at the reflective tip of the sensor and is thereby cast onto a screen covered with graph paper. There, the oscillation of the sensor can be seen as an oscillating point or (due to the fast oscillation) as a laser stripe.

Conducting the experiment: The sensor (the thin steel attached to the CD stripe) is clamped on one side and inserted in the coil. The laser pointer is adjusted in a way that the beam reflects off the tip of the CD strip and a laser dot is visible on the screen. The current generated by the function generator should be between 10 and 100 mA. Then, the frequency of the AC voltage can be increased from 1 Hz upwards. Depending on the sample, large deflections can still be found at 200 Hz. By controlling the frequency, one can look for the natural resonance point of the sample. At this point, the oscillation of the sample will be very large. At a frequency that is not the resonance frequency of the sensor, the current can be increased up to 100 mA - a deflection of the oscillating laser spot is visible.

The oscillating laser point can be seen as a line - the slow motion function of smartphones can be used for better visualization.

After having seen this experiment conducted by the supervisor, the students shall explain the experiment.

Explaining the Experiment (15 min)

To fully explain this experiment, there are more variables needed than those already mentioned to the students. Since it would be too much to expect the students to be able to explain the experiment without any further information, they are given cards with hints and questions to get them on the right track.

To record their explanation, they can document the deflection of the bending beam sensor on their worksheet (task 2).

Conducting Research with Help of the Experiment (20 min)

After having understood the model experiment set-up, the students shall use the set-up to answer the following questions:

1) Determine in which direction the bending beam sensor is deflected by using the laser pointer.

2) How does the strength of the magnetostriction change with increasing (and decreasing) current strength?

3) What effect does the width of the magnetostrictive material have on the behavior of the sensor?

4) Which properties of the magnetostrictive material could also influence the sensor behavior?

For this purpose, they are given different sensor samples. They can repeatedly determine the natural resonance frequency by adjusting the frequency and compare the samples with each other on the basis of the values determined. In addition, they can increase or decrease the current and observe the effects of this. They summarize their observations regarding the relationship between the current strength and the deflection of the sensor, i.e. the strength of the magnetostrictivity, in a sentence on their worksheet: The higher the current strength, the stronger the magnetic field. The stronger the magnetic field, the stronger the magnetostrictive effect.

In the end, the learners receive two cards:

**Magnetostriction**<br>**magnetic → mechanical**	**Bending Beam Principle**<br>**mechanical → mechanical**
