# Supplementary material for: Teaching Magnetoelectric Sensing to Secondary School Students—Considerations for Educational STEM Outreach
Source: Sensors (Basel). 2021 Nov 5;21(21):7354. doi: 10.3390/s21217354 (PMC8588487; doi:10.3390/s21217354)
Supplement: Supplementary file 1 [file sensors-21-07354-s001.zip › Station 3/Worksheet_station3.pdf]

Magnetostriction

1. *Before the Experiment*

Definition of **magnetostriction:**____
____
____

Task 1

Draw a sketch that shows how a bending beam sensor works.

Outside of a magnetic field:

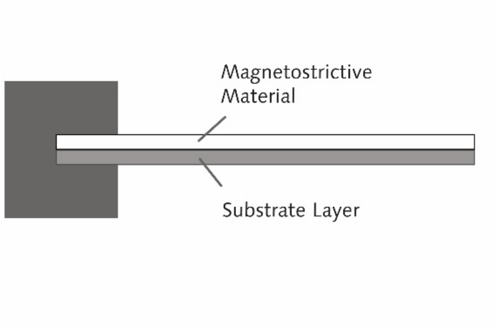

Inside of a magnetic field:

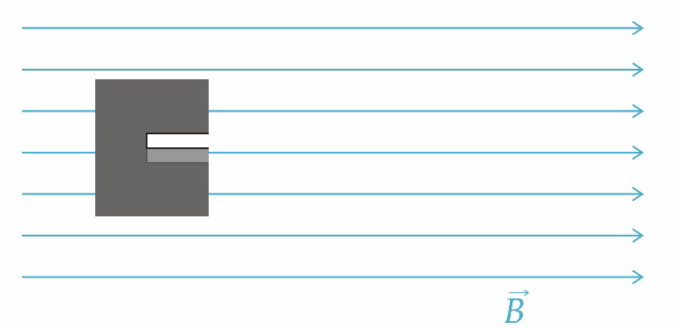

2. *During the Experiment*

Task 2

For each sample, write down its properties (length, width and thickness of the respective materials, etc.). Then, write down the frequency values at which you observed an unusually high excursion. These values are the values of the natural resonances of the sample.

	Properties of the Sample	Natural Resonance Frequency
**Sample 1**		
**Sample 2**		
**Sample 3**		

Task 3

Investigate the relationship between the strength of magnetostriction and the current strength.
Summarize your observations in the following sentence:

The higher the current strength, the ____.

3. After the Experiment

Task 4

Note, which properties of the magnetostrictive material influence the behavior of the sensor and how they influence it.

____
____
____
____
____
____
