# Supplementary material for: Teaching Magnetoelectric Sensing to Secondary School Students—Considerations for Educational STEM Outreach
Source: Sensors (Basel). 2021 Nov 5;21(21):7354. doi: 10.3390/s21217354 (PMC8588487; doi:10.3390/s21217354)
Supplement: Supplementary file 1 [file sensors-21-07354-s001.zip › Station 4/Instructions for supervisors_station4.pdf]

<u>**Station 4 – Building a ME sensor**</u>

Brief Overview:

At this station, students are to combine the knowledge they gained at the previous stations by building their own sensor. The self-built sensor is then tested and compared with other groups. They reflect on what worked well and possible improvements to the sensor.

Duration: 45 minutes

Participants: 3 - 5 school students (one group)

Time table:

Time	Topic	Short description
10 min	Theoretical introduction	Combining basic knowledge of previous stations for insights into the ME sensor principle and sensor design
25 min	Experiment	Building and testing the ME sensor model
10 min	Analysis and transfer	Comparing sensors among the groups<br>Reflecting on the built sensors and comparing them to the sensors of the CRC

Theoretical Introduction (10 min)

At this point, the students have accumulated all of the basic knowledge they need to carry out the next experiment: they learned about the principle of magnetostriction, piezoelectricity and the bending beam sensor. They also learned about the application context for which a sensor is to be developed: for the detection of biomagnetic fields. They are now to combine these elements of knowledge.

They are given the task of coming up with a sensor design that converts magnetic signals into electrical signals. If they need help, they shall repeat which materials and which sensor design they have learned about. In particular, they can recapitulate which types of signals can be converted by the materials they got to know. For this purpose, they can use the cards they have collected (Figure 1).

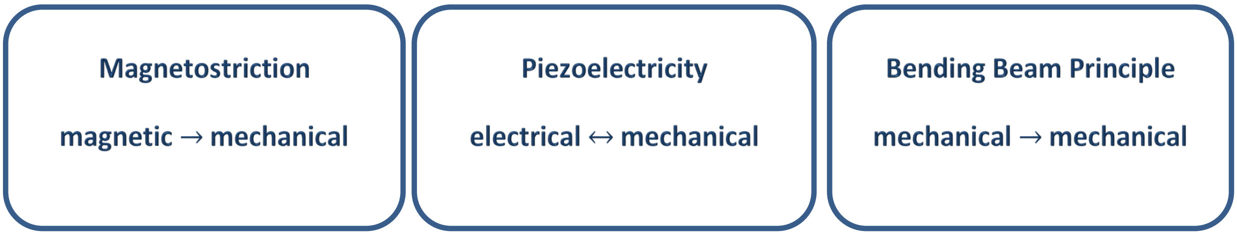

Figure S1. Cards students collected at station 2 and 3.

The end result should be the following: The magnetic signal can be converted into a mechanical signal with the help of magnetostriction. This mechanical signal can be passed on to the piezoelectric material by the bending beam principle. The piezoelectric material converts the mechanical signal into an electrical signal. The process is summarized in Figure 2.

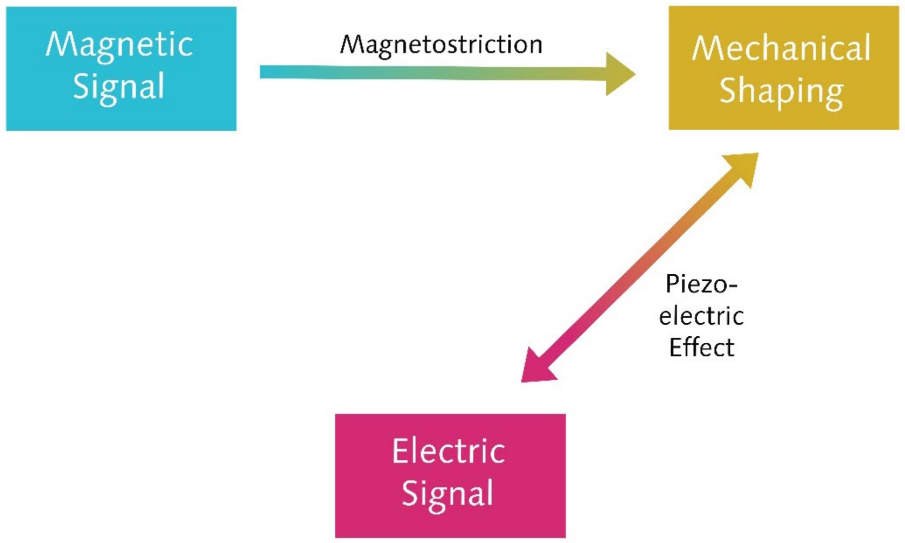

Figure S2. Simplified, schematic representation of the sensor principle.

With or without this intermediate step, the students should think about how they can build a sensor that converts magnetic signals into electrical signals. They should complete this task on their own if possible. If they cannot think of a possible sensor design, they can be reminded of the sensor they got to know during the magnetostriction model experiment at station 3.

Experiment (25 min)

The materials that the students can use for their ME sensor design are limited: a CD strip that serves as a carrier layer, a piezo element and thin steel in different sizes.

One possible setup for the sensor would be to add a piezoelectric material to the sensor from the experiment at station 3. Apart from that, many more sensor designs are possible. Before students can build their self-designed sensor, they have to discuss their plan with a supervisor. The supervisor has to approve of the plan. If they do not approve of the group's plan because they think that the presented sensor design will not work, they give individual tips. Afterwards, the supervisor gives instructions on how to process the materials.

Instructions on how to process the materials: The surfaces to be agglutinated should be thoroughly sanded beforehand and touched as little as possible with bare hands, as the resulting greasy film would counteract the optimum adhesive bonding of the materials. After the superglue has been applied, the materials should be pressed firmly together for a short time. When attaching the piezo element, care must be taken not to damage the piezoelectric layer. A piece of hard foam can be used for this purpose.

Testing the sensor: The finished sensor is clamped into the measuring device: clamped on one side of the CD stripe and inserted in a coil at the other side of the CD stripe. The voltage pickups of the piezo element are connected to an oscilloscope using crocodile clips. For the coil, a voltage of 10 V and a current of about 50 mA is set with a frequency generator. The frequency is then increased in small steps from 1 Hz to 160 Hz in order to find the resonance frequency of the sensor.

Analysis and Transfer (10 Min)

Students can compare their results with other groups and discuss sensor setups. In the case of non-functioning sensors, students can try to think of possible sources of errors. If there is enough time, groups receive the opportunity to build an optimized sensor.

The class reflects on how realistic the replicated sensor is and thinks about possible ways to improve the sensor. An outlook on the research of the CRC is given.
